# Quercetin Inhibits the Proliferation of Glycolysis-Addicted HCC Cells by Reducing Hexokinase 2 and Akt-mTOR Pathway

**DOI:** 10.3390/molecules24101993

**Published:** 2019-05-24

**Authors:** Hongyan Wu, Lanlan Pan, Cuixiang Gao, Hongtao Xu, Yanping Li, Lihu Zhang, Linwei Ma, Li Meng, Xiulan Sun, Hongbing Qin

**Affiliations:** 1 Institute of Biomedical Technology Co., Ltd., Jiangsu Vocational College of Medicine, Yancheng 224005, China; why20133055@163.com; 2 Department of Pharmacology, School of Basic Medical Sciences, Nanjing Medical University, Nanjing 211166, China; 3 Jiangsu Vocational College of Medicine, Yancheng 224005, China; panlanlan930@163.com (L.P.); gcx31512000@163.com (C.G.); xht800703@163.com (H.X.); liyanping502@163.com (Y.L.); zlh800927@163.com (L.Z.); malinwei2000@163.com (L.M.); li_972@163.com (L.M.)

**Keywords:** quercetin, hepatocellular carcinoma (HCC), glycolysis, hexokinase-2(HK2)

## Abstract

Increased glycolysis in tumor cells is associated with increased risk of tumor progression and mortality. Therefore, disruption of glycolysis, one of the main sources of cellular energy supply, can serve as a target for suppressing tumor growth and progression. Of note, hexokinase-2 (HK2) plays vital roles in glucose metabolism. Moreover, the expression of HK2 alters the metabolic phenotype and supports the continuous growth of tumor cells, making it an attractive target for cancer therapy. Quercetin (QUE), a bioactive flavonoid, has a profound anti-tumor effect on hepatocellular carcinoma (HCC), but the precise underlying mechanism of this effect is unclear. In the present study, we reported that QUE inhibited the proliferation of HCC cells that relied on aerobic glycolysis. We further found that QUE could decrease the protein levels of HK2 and suppress the AKT/mTOR pathway in HCC cells. In addition, QUE significantly restrained the growth of HCC xenografts and decreased HK-2 expression in vivo. Taken together, we have revealed that QUE suppresses the progression of HCC by inhibiting HK2-dependentglycolysis, which may have a promising potential to be an effective treatments for HCC, especially for those patients with high HK2 expression.

## 1. Introduction

Hepatocellular carcinoma (HCC), one of the most frequently occurring malignancies with highly abnormal metabolic status, has become the second leading cause of cancer-related deaths worldwide [1,2,3]. Currently, surgical resection, orthotopic liver transplantation, and radiofrequency thermal ablation are the mainstay of curative treatment options for HCC patients [4]. However, the long-term outcome after such treatment is still poor. Thus, better innovative strategies or paradigms for HCC treatment are urgently needed. One of the metabolic features of HCC cells is that the glucose metabolism always terminates in pyruvate and bypasses the oxidizing with the Krebs cycle, which can convert pyruvate to lactate acid under sufficient oxygen. [5,6]. This phenomenon, commonly known as “Warburg effect”, has become a new marker of cancer cells [7,8]. HK2, which is overexpressed in numbers of human cancer, is the rate-limiting enzyme catalyzes the first irreversible step of glycolysis [9,10]. Recently, several studies indicate that HK2 is overexpressed, and promotes tumorigenesis of HCC. Furthermore, HK2 expression in HCC is correlated with poor prognosis and malignant phenotype [11,12,13]. These results suggest that HK2 may play an essential role in HCC carcinogenesis and the screening of agents that target this molecule can provide an optional therapeutic approach.

Quercetin (QUE), which has diverse effects including antioxidant, anti-inflammatory, vasodilatory and anticancer effects, is a natural multifunctional flavonoid [14]. Accumulated studies have implied that QUE can exert antitumor activities at different stages of tumor initiation and progression [15,16,17]. Moreover, to the anti-tumor activities of QUE are well characterized, including suppression of various tumorgenesis-related transcription factors, inhibition of the activities of protein kinases, down-regulation of the expression of genes associated with cell cycle progression, angiogenesis, anti-apoptosis and metastasis, and so on [18,19]. Recently, QUE was proved to be able to inhibit cell glycolysis the progression in breast cancer [20,21]. Besides, novel evidence has shown that QUE can downregulate the PI3K-Akt1-p53 pathway, decrease glycolytic metabolism and cause growth suppression in Dalton’s lymphoma [22]. However, it is unclear whether QUE can inhibit the proliferation of HCC cells by affecting the glycolysis and the related pathways. In this study, we aimed to verify whether QUE could impair the eHCC metabolism and its dependency on on tumor growth. In the present study, we confirmed the therapeutic potential of QUE by disruption of HK2-mediated HCC metabolism.

## 2. Results

### 2.1. The Inhibitory Effect of Quercetin (QUE) on Proliferation Was Correlated to the Suppression of Glycolysis in Hepatocellular Carcinoma (HCC)

**Figure 1:**
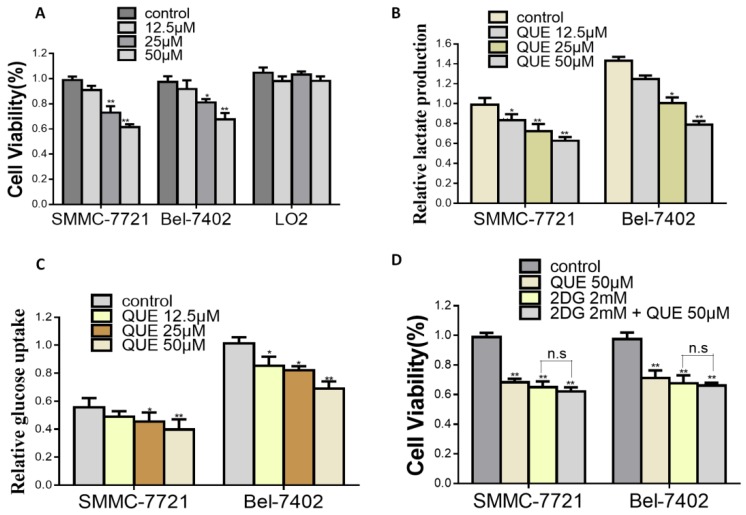
Inhibitory effects of quercetin (QUE) on cell viability and glycolysis in hepatocellular carcinoma (HCC) cells. (**A**) HCC cells were cultured with or without QUE (12.5, 25, and 50 µM) for 24 h and cell viability was detected through MTS[3-(4,5-diethylthiazol-2-yl)-5-(3-carboxymethoxyphenyl)-2-(4-sulfophenyl)-2H-tetrazolium, inner salt] assay. (**B**,**C**), The levels of lactate production (**B**) and glucose uptake (**C**) from HCC cell lines (SMMC-7721, Bel-7402) in the absence or presence of QUE at indicated concentrations for 24 h. (**D**) HCC cells were cultured in the presence of QUE, or 2DG or 2DG plus QUE at indicated concentrations for 24 h. Cell viability were detected. All experiments were performed in triplicate with similar results (* P < 0.05; ** P < 0.01 versus control; n.s means no significance).

Based on previous studies, Bel-7402 and SMMC-7721 cells, which displayed a high aerobic glycolysis rate, were selected as cell models in the following experiments [12,13]. In accordance with previous results [23,24], our data also showed that cell proliferation in Bel-7402 and SMMC-7721 was effectively inhibited albeit to varying degrees after QUE treatment for 24 h (Figure 1A). In view of the fact that glycolysis is the main energy provider for tumor progression, we then set out to explore the effect of QUE on the glycolysis of HCC cell lines. As shown in Figure 1B,C, glucose uptake and lactate production of SMMC-7721 and Bel-7402 decreased in a dose-dependent manner after QUE treatment. Recent studies have shown that QUE can inhibit the glycolysis of cancer cells, thereby inhibiting the progression of multiple cancers [14,20,21]. Therefore, we further examined whether the inhibition of QUE on cell growth was related to the inhibition of glycolysis by hypothesizing that glycolysis inhibitors might attenuate the growth inhibition of QUE on aerobic glycolytic HCC cells. Intriguingly, although cell proliferation assays show that 2-DG, a synthetic glycolysis inhibitor, distinctly restrains cell growth, this inhibitory effect was not increased upon co-treatment with QUE and 2-DG (Figure 1D). Altogether, these results indicate that QUE-mediated inhibition of HCC cell proliferation is likely to be exerted through inhibition on glycolysis.

### 2.2. HK2 is Essential for QUE-Suppressed HCC Glycolysis and Proliferation

**Figure 2:**
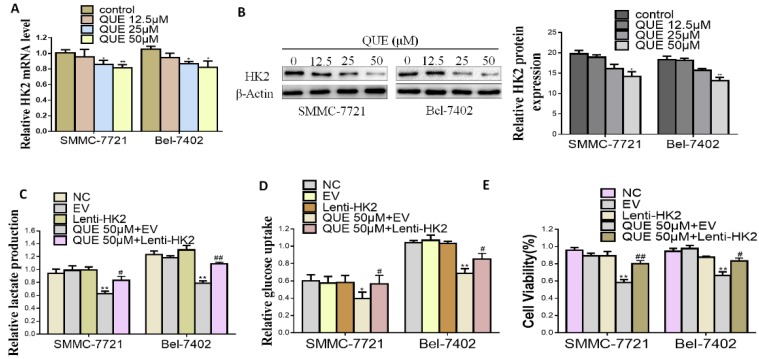
HK2 is essential for QUE-suppressed HCC cells glycolysis. (**A**,**B**) real-time polymerase chain reaction (PCR) and Western blot analyses of the effect of QUE on the level of HK2. β-Actin was used as the invariant control (**C**–**E**) SMMC-7721 and Bel-7402 were stably transfected with Lenti-HK2 with or without QUE 50 µM for 24 h. At the time points indicated, the following measurements were performed: lactate production (**C**), glucose consumption (**D**), cell proliferation rate (**E**). Representatives were from three parallel experiments (* P < 0.05; ** P < 0.01 vs. *NC group; ^#^ P < 0.05; ^##^ P < 0.01* vs. EV group treated QUE). NC: negative control; EV: empty vector.

HK2, which participates in cell growth regulation and is unregulated in multiple cancers, is the first important rate-limiting enzyme in glycolysis [9]. Next, we measured whether QUE had any influence on the expression of HK2 in HCC cells by quantitative reverse-transcription polymerase chain reaction (qRT-PCR) and Western blotting assays. As shown in Figure 2A,B, after QUE treatment, HK2 mRNA and total protein expression level significantly decreased in a dose-dependent manner. To further study the role of HK-2 played in QUE-mediated activities, SMMC-7721 and Bel-7402 cells stably overexpressing HK2 (Figure S1) were treated with QUE, which significantly attenuated its inhibitory effect on glucose uptake, lactate production and cell proliferation. As shown in Figure 2C,D, analysis of hallmarks of glycolysis showed that the inhibitory effect of QUE was totally decreased in HK2 overexpressing groups rather than in empty vector (EV) groups. The same was true for cell proliferation rate (Figure 2E). Altogether, the results demonstrate that HK2 is critical for the QUE-inhibited glycolysis and cell proliferation in HCC cells.

### 2.3. QUE Suppressed Glycolysis through Akt-mTOR Pathway-Mediated HK2 Regulation in HCC Cells 

**Figure 3:**
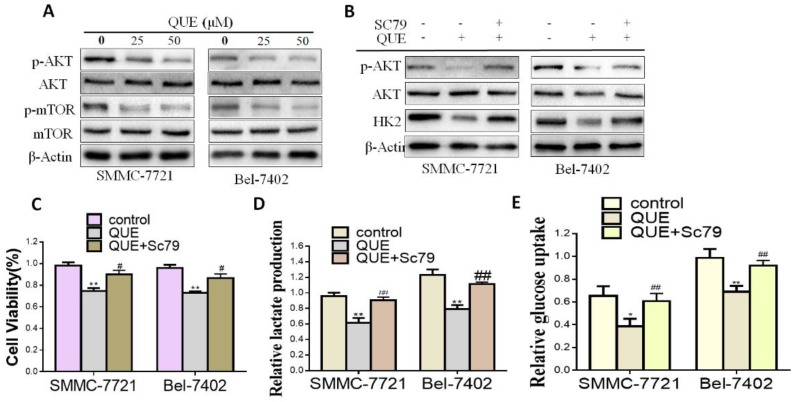
QUE suppressed HCC cells glycolysis through Akt-mTOR pathway. (**A**) Western blot analyses of the effect of QUE on the expression of p-Akt/Akt, p-mTOR/mTOR. β-Actin was used as the invariant control. (**B**) HCC cells were cultured with or without SC79 (5 μg/mL) for indicated time after QUE (50 μM) treatment and then the following measurements were performed: cell proliferation rate (**C**), lactate production (**D**), glucose consumption. (**E**) Representatives were from three parallel experiments (* P < 0.05; ** P < 0.01 vs. control group; *^#^ P < 0.05; ^##^ P < 0.01* vs. QUE treatment group).

In order to further determine the mechanism of QUE modulation of HK2 expression level, we focused on the Akt-mTOR pathway, which regulates a wide variety of cellular processes including cancer cells glucose metabolism [25,26]. As shown in Figure 3A, compared with the control group, QUE treatment effectively inactivated the Akt-mTOR pathway by inhibiting the rates of p-Akt /AKT and p-mTOR/mTOR. To further clarify whether the Akt-mTOR pathway was involved in the inhibition of HK2 by QUE, Akt phosphorylation activator (SC79, a compound for research tool) were used (Figure 3B) [27]. As shown in Figure 3C–E, SC79 treatment attenuated QUE-inhibited HCC cell proliferation (Figure 3C) and reversed the glycolysis inhibitory effect of QUE (Figure 3D,E). Furthermore, HK2, the rate-limiting enzyme catalyzing the first important irreversible step of glycolysis were dramatically elevated, suggesting that the disruption of Akt-mTOR pathway is responsible for HK2 expression and resulted in HCC glycolysis and proliferation inhibitory effect of QUE.

### 2.4. QUE Inhibits Tumor Growth and HK2 Expression in a Xenograft Mouse Model

**Figure 4:**
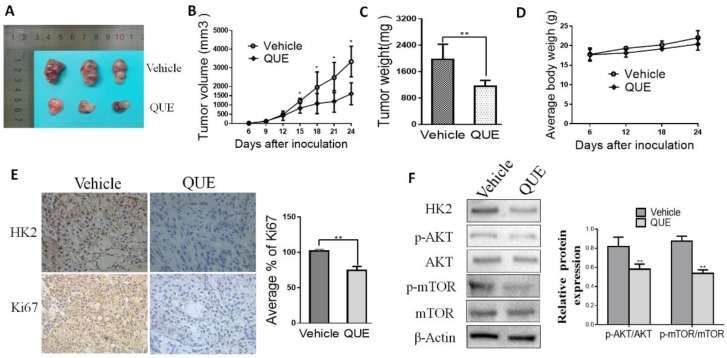
QUE inhibits tumor growth in SMMC-7721 xenograft mouse models. Nude mice with SMMC-7721 xenograft were randomly divided to groups and then 50 mg/kg QUE was administrated. The tumor area was measured every 3 days and the tumor volume and tumor weight was calculated as described in Materials and Methods. (**A**) photograph of tumors in vehicle and QUE-treated group; (**B**) tumor growth curve in vehicle and treated group; (**C**) tumor weight in vehicle and QUE group; (**D**) during the treatment period, the body weight of mice was measured once a week to determine the effect of QUE. (**E**) Immunohistochemical staining examination of Ki67 and HK2 in tumor sections from vehicle or QUE treated group. All panels are of the same magnification. The histogram on the right represents the average percentage of Ki67 expression. (**F**) Expression of HK2, Akt and phosphorylated Akt, mTOR and phosphorylated mTOR in harvested tumors. The tumors lysates were harvested and phosphorylation of indicated proteins was detected by western blotting. β-Actin were used as internal standards (* P < 0.05; ** P < 0.01 vs. Vehicle).

According to the results of in vitro experiments, we have further studied the effects of QUE on the growth of tumors and the expression of HK2 by using a human HCC xenograft. Giving 5-week-old female nude mice a subcutaneous injection of SMMC-7721 cell lines created a xenograft mouse model, as mentioned previously [28]. Our results showed that QUE significantly reduced the size of tumors in a SMMC-7721 xenograft model (Figure 4A–C). Weights of all mice increased normally among vehicle control or QUE group (Figure 4D) Moreover, immunohistochemical and Western blot analyses of QUE treated SMMC-7721 xenograft tumors was performed to determine the expression level of HK2. Data showed that HK2 were remarkedly down regulated in the QUE treated group rather than in the vehicle group (Figure 4E,F). As a well-known marker of cell proliferation potential, Ki-67 was also detected by immunohistochemistry staining. As shown in Figure 4E, compared with the Ki67 in the vehicle group, Ki67 in QUE group was substantially decreased. Furthermore, we found that phosphorylated Akt and mTOR was dramatically inhibited in QUE treated group (Figure 4F). The above results clearly indicate that QUE suppresses the progression of HCC by inhibiting HK2 mediated glycolysis in vivo.

## 3. Experimental Section

### 3.1. Antibodies and Reagents

In this study, the regents we used includes Quercetin (QUE, purity 98.0%) purchased from Yuanye Bio-Technology Co, Ltd. (Shanghai, China) and 2-Deoxy-D-glucose (2DG) (Tocris Bioscience, Bristol, UK); the antibodies we used in this research includes HK2 (Proteintech, Wuhan, China); Akt, p-Akt, mTOR, mTOR (Cell Signaling Technology, Danvers, MA, USA); β-Actin (Sigma, St Louis, MO, USA). The fluorescein isothiocyanate (FITC)-conjugated secondary antibodies and all horseradish peroxidase-conjugated secondary antibodies were obtained from Cell Signaling Technology (Danvers, MA, USA).

### 3.2. Cell Culture and Animals

Human HCC cell lines SMMG-7721, BEL-7402, and normal hepatic cell line LO2, which were purchased from the Cell Bank of Type Culture Collection of the Chinese Academy of Sciences (Shanghai, China), cultured in Dulbecco’s modified eagle medium (DMEM) (Hyclone, Pittsburgh, USA) with10% fetal bovine serum (FBS; Cellmax, Beijing, China), 100 μg/mL streptomycin and 100 U/mL penicillin and grown at 37 °C in a 95% air and 5% CO_2_ humidified atmosphere, is one of our most important cells represented in our study. Twelve male BALB/c nude mice (5~6 weeks old) is the most important animals we used in our research, which were purchased from the Experimental Animal Center of Nanjing Medical University and fed under specific pathogen-free conditions. 

### 3.3. Cell Proliferation Assays

CellTiter 96^®^ A Queous One Solution Cell Proliferation Assay (MTS) (Promega, Madison, WI, USA) is used in this paper to measure the influence of QUE on proliferation of cell. To be short, we seeded HCC cells in 96-well plates (4 × 10^4^ cells/well) and put it in a serum-starved environment in DMEM for 24 h. Then we put cells in various concentrations of QUE, respectively, according to indication, and incubated cells with the MTS reagent (5 mg/mL, 10 μL/well) for 4 h. Finally, we used Synergy TM 2 Multi-function Microplate Reader (Bio-Tek Instruments, Winooski, Vermont, VT, USA) to determine the optical density (OD) at 490 nm due to the instructions we mentioned above [28]. A microplate reader can measure the absorbance at 490 nm. Each experiment was conducted five times.

### 3.4. Glucose Uptake and Lactate Production Assays

As we described previously, glucose uptake was measured by glucose (Hexokinase) assay kit (Nanjing Jiancheng Bioengineering Institute, Nanjing, China) as per the manufacturers’ explanatory memorandum [29], and lactate production was performed by lactate assay kit (Nanjing KeyGen Biotech Co., Ltd., Nanjing, China). All the measurements were standardized for cell counts and the experiment was carried out.

### 3.5. RNA Isolation and Quantitative Real-Time Polymerase Chain Reaction (qRT-PCR) 

We applied TRIzol reagent (Invitrogen, Carlsbad, CA, USA) to isolating total RNA. In a PrimeScript RT reagent kit (TakaraBio, Tokyo, Japan), we synthesized first-strand of cDNA with 1μg of total RNA. and conducted the iQ5 real-time detection system and IQTM SYBR Green supermix (Bio-Rad Laboratories, Hercules, CA, USA) to analyze qRT-PCR. The following primers were applied for qRT-PCR: sequences of primers for human HK2 are forward 5′-TCGG ACAGGCCACAGC AGTG-3′ and reward 5′-AGCTCTG TGGCGCAGGCATG-3′, forward 5′-GACCGTCTGAGTGGGAAG-3′ and reward 5′-TTACCAGAAAGGGCACCAG-3′, for human mTOR, (forward)5′-AGAAGGC TGGGGCTCATTTG-3′ and (reward) 5′-AGGGGC CATCCACAGTCTTC-3′ for human GAPDH. We put 2 pM of forward and reverse primers and 7.5 μL of SYBR Green I dye master mix (Quanta) together in reaction mixtures at 50 °C and 95 °C (10 min each) initially, and then we put the mixture at 95 °C (15 s) and 60 °C (1 min) for 40 cycles. The levels of mRNA were normalized in relevance to GAPDH and we conducted the 2^-ΔΔCT^ method to determine mRNA abundance. Experiments were performed three times.

### 3.6. Western Blotting

We extracted and prepared total lysates from treated HCC cells and tumor tissue. We used a BCA (bicinchoninic acid) assay kit (Pierce, Rockford, IL, USA) to detect the protein levels, and we separate equivalent amounts of protein (40 μg/lane) by SDS (sodium dodecyl sulfate) polyacrylamide gel, and used 5% skim milk in Tris-buffered saline containing 0.1% Tween 20 to transfer it to PVDF (polyvinylidene fluoride) membranes (Millipore, Bedford, MA, USA). And then we used primary antibodies to incubate membranes, and use appropriate secondary antibodies to conjugate to horseradish peroxidase. We used a chemiluminescence reagent (Millipore, Burlington, MA) by the Bio-Rad Universal Hood II DOC Electrophoresis Imaging Cabinet to visualize immunoreactive bands and use an antibody against β-Actin to confirm equivalent loading respectively. The proposed blots are representative of three independent experiments.

### 3.7. HK2 Lentivirus Vector Transfection

We plated aliquots of 1 × 10^5^ HCC cells in 1 mL of antibiotic-free medium on 6-well plates. And we incubate cells for 24 h and washed them with PBS and then added serum-free medium in them. Control lentiviral vector and lentivirus vector pCDH-HK2 (Genechem Co., Ltd., Shanghai, China) were used following the specifications and instructions. We used empty vector or pCDH-HK2 to infect target cells in the environment of 8 μg/mL polybrene (Sigma-Aldrich). We put cells in fresh medium and made it grow for an additional 36–48 h the next day. Western blotting is used to analyze the transduction efficiency and then we analyze the character of sorted cells, which are used in further research.

### 3.8. Animal Experiments and Ethics Statement 

All animal studies were reviewed and approved by the Institutional Animal Care and Use Committee of Nanjing Medical University. Nude mouse tumor xenograft assay was performed as we mentioned above [28]. Briefly, we put HCC cells (1 × 10^7^ in 0.1 mL of HBSS (Hank′s Balanced Salt Solution)) above the serum free-RPMI/Matrigel mixture (2:1 vol) and injected it in the subcutaneous tissue into the lateral ventral region of mice. After 6 days, we divided mice into 2 groups randomly (1 groups, 6 mice): (1) The control group mice were injected with normal saline. (2) We injected the mice in group QUE intraperitoneally with 50 mg/kg QUE twice a day for 18 days. Calculate the volume of tumors every 3 days after injection according to the following standard formula: tumor volumes (mm^3^) = width^2^ × length/2. We recorded the mass and volume of the final tumors at 24 days and then executed the mice. We harvested and fixed the tumors in 4% paraformaldehyde and stored them, which are used for next experiments at room temperature.

### 3.9. Immunohistochemistry

Due to our previous studies, we conducted an immunohistochemical assay using following steps [29]. Briefly, we deparaffinised and rehydrated formalin-fixed paraffin-embedded sections (4 μm) and washed it with PBS. We quenched endogenous peroxidase and sliced with skimmed milk. Then we applied primary antibodies against Ki67 and HK2 at room temperature for 1 h. After washing with PBS, we incubated a peroxidase-labelled dextran polymer by using secondary antibodies against mouse and rabbit IgG for 1 h. DAB (Beyotime Institute of Biotechnology, Haimen, China) systems were used for detection as described earlier [30].

### 3.10. Statistical Analysis

We use SPSS23.0 software analyze Data, which were expressed as mean ± SD. After analysis of variance (ANOVA), we determine the significance of difference by Student-Newman-Keuls *t-test*. And values of *p < 0.05*, which was considered to be statistically significant.

## 4. Discussion

A large number of studies have shown that tumor cells obtain energy from “Warburg effect” and sustain this for proliferation. Meanwhile, increased glucose uptake can make sure that energy can be supplied to compensate for the shortage of ATP generated by glycolysis all the time [31]. To this end, blocking the glycolysis of tumor cells to cut off energy supply has become a research focus in the field of cancer therapy nowadays [32].

Previous research has shown that decreased glycolytic metabolism in breast and in Dalton’s lymphoma can cause the reduced progression induced by QUE [14,20,21]. Although the anticancer properties of QUE are well known, the relationship between QUE and cancer metabolism is poorly understood, including the role of key metabolic enzymes such as HK2. In the present study, we have shown that antiproliferative effects of glycolytic inhibitors(2DG) were not enhanced when the aerobic glycolytic HCC cells were treated together with QUE (Figure 1D), indicating that cell growth inhibition was probably correlated to QUE-inhibited aerobic glycolysis. In addition, we also found for the first time that the glycolysis and growth of HCC cells were significantly inhibited with the decrease of HK2 after QUE treatment (Figure 2A,B). Further studies have shown that the suppression induced by QUE was substantially weakened after overexpression of HK2, suggesting HK2 is essential for QUE to exert its activity in HCC (Figure 2C–E).

The first rate-limiting enzyme of glycolysis is translocated to the outer membrane of mitochondria and interacts with the pore-like protein voltage-dependent anion channel (VDAC) to inhibit apoptosis, which is another mechanism for HK2 promoting the survival of cancer cell [33,34,35]. In this study, we only focused on the effects of QUE on inducing glycolysis and growth inhibition in HCC cells, however, we can’t rule out the possibility of QUE on down regulating HK2 and the likelihood of it impairing the balance between cell death and survival.

It is well known that Akt-mediated signaling pathways including cell survival, migration, growth and metabolism are critical steps for numbers of cellular processes [27,36]. Akt is involved in the regulation of cellular survival through phosphorylation of downstream substrates that directly or indirectly control the metabolic machinery. Of note, Akt-mTOR pathway has been indicated as a metabolic regulatory center of cancer [8,37,38]. Recently, studies have documented that Akt-mTOR pathway could modulate HK2, a pivotal role in the procession of cancer metabolism, leading to tumor rapid growth, even in the presenceof oxygen [38]. Reducing HK2 expression can impede glycolysis and thus restrain cancer progression as well as HCC [12,13,38]. In the present study, further investigation indicated the inhibition of glycolysis was closely associated with the effect of QUE on the Akt-mTOR pathway (Figure 3). Here, we found with the inhibition of HK2 expression after QUE treatment, the phosphorylation of mTOR and Akt also reduced. On the one hand, we confirmed that SC79, the Akt phosphorylation activator, can moderately restore cell growth and glycolysis after QUE treatment (Figure 3C–E). As we can see, the inhibition of QUE on glycolysis by regulating HK2 in HCC can be reflected better in these results. Nevertheless, even though we can use SC79 treatment to induce Akt phosphorylation in cells treated by QUE, the level of phosphorylation Akt was less than that in the control group, suggesting that other factors or potential targets besides the Akt signaling pathway presumably were also involved in the inhibition of QUE-mediated glycolysis. Indeed, the regulation of HK2 is complicated in cancer cells. Although the potential mechanisms of the HK2 deregulation in cancer cells has not yet been fully elucidated, c-Myc, as well as HIF-1α and p53 have been proved to be involved in HK2 and HK2-mediated glycolysis demodulation [39,40,41]. Hence, an exact molecular mechanism of the effect of QUE on the Akt-mTOR pathway warrants further elaborate investigation.

In conclusion, this study indicated that glycolysis is involved in QUE-mediated anti-tumor activity on HCC in vitro and in vivo after being studied through the glucose metabolism pathway. For the first time, we identified the reduction of HK2 was an important basic mechanism for QUE to play its role in HCC cell growth and metabolism, which provided a novel theoretical basis for preclinical clinical research for QUE in the application of HCC treatment.

## Supplementary Materials

The following are available online. Figure S1: Transduction efficiency of HK2 overexpression in HCC cells.
