# Supplementary figures and images for: Quercetin Inhibits the Proliferation of Glycolysis-Addicted HCC Cells by Reducing Hexokinase 2 and Akt-mTOR Pathway
Source: Molecules. 2019 May 24;24(10):1993. doi: 10.3390/molecules24101993 (PMC6572074; doi:10.3390/molecules24101993)

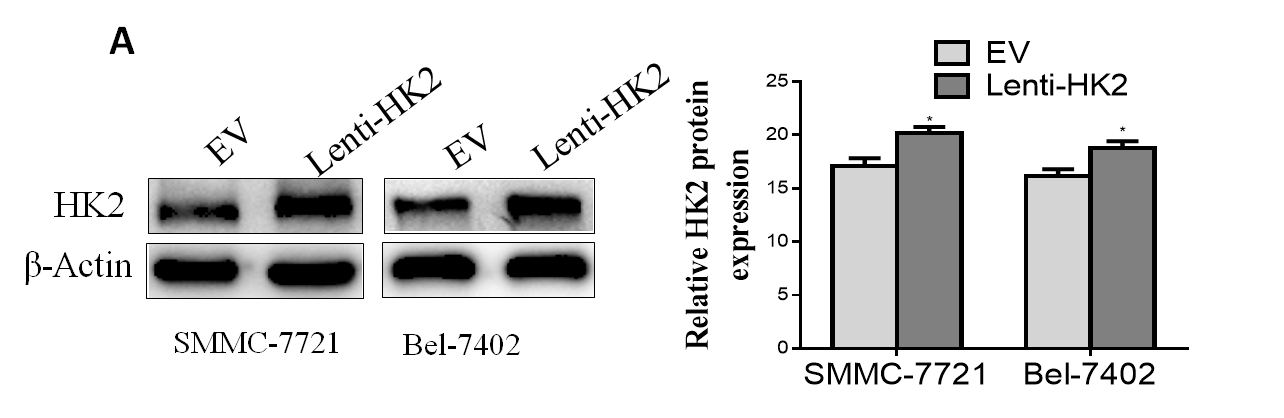

Supplement: Supplementary file 1 [file molecules-24-01993-s001.zip › molecules-504320-SI.tif]
